# Oral health outcomes following early-life exposure to war-related hardship: a cross-sectional study

**DOI:** 10.1007/s00784-026-06821-y

**Published:** 2026-03-17

**Authors:** Guy Tobias, Jonathan Mann, Avraham Zini, Harold David Sgan-Cohen

**Affiliations:** Faculty of Dental Medicine, Department of Community dentistry, Hebrew University of Jerusalem, Hadassah Medical Center, Jerusalem, Israel

**Keywords:** Dentures, Oral health, Holocaust, Trauma

## Abstract

**Background:**

The Holocaust was a uniquely traumatic historical event marked by prolonged periods of starvation, extreme physical and psychological stress, and deprivation of basic human rights. While previous research has explored the general health consequences of Holocaust exposure, the long-term effects on oral health have received limited attention. Oral health is an essential component of overall well-being, with edentulism (complete tooth loss) recognized as a marker of severe oral disease and functional impairment in older adults. This purpose of this study was to assess the long-term impact of Holocaust exposure on oral health outcomes, specifically the prevalence of complete edentulism, among elderly Jewish Israelis more than six decades after World War II.

**Methods:**

A cross-sectional analysis was conducted using data from a nationallyrepresentative Israeli survey of adults aged 65 and older, carried outbetween 2005 and 2006. The sample included 1,459 Jewish participants,of whom 396 were identified as Holocaust survivors. Data were collectedvia structured in-home interviews and, for a subset, clinical oralexaminations. Multivariate logistic regression was used to identifypredictors of having two full dentures, serving as a proxy for completetooth loss.

**Results:**

Holocaust survivors exhibited significantly higher rates of complete edentulism compared to the control group (56.3% vs. 45.7%, p < 0.01). After adjusting for confounders, factors independently associated with having two full dentures included older age, smoking history, recent dental visits, Holocaust exposure, lower religiosity, and caregiver dependence. The final model explained 22.2% of the variance in denture status.

**Conclusion:**

Holocaust survivors exhibited poorer oral health compared to their peers,with several contributing factors identified in the analysis.These findings highlight the need for tailored dental and health servicesfor Holocaust survivors and other trauma-exposed populations, andunderscore the long-term oral health implications of early-life adversity.

## Background

In January 1933, the Nazi Party came to power in Germany, with Adolf Hitler appointed as Chancellor. The Nazis quickly dismantled democratic institutions and established a dictatorship in the Third Reich [1]. Early on, in April 1933, the regime initiated its first anti-Jewish action by organizing a nationwide boycott of Jewish businesses [2]. This economic persecution was soon accompanied by cultural repression [3]. Over the following years, the Nazi regime’s policies grew increasingly violent and oppressive. This also facilitated the confiscation of Jewish property [4], life were characterized by extremely adverse conditions. Severe overcrowding was commonplace [5, 6], and chronic food shortages led to vitamin and mineral deficiencies [7, 8]. Poor sanitation and hygiene contributed to outbreaks of infectious diseases such as typhoid and tuberculosis [9]. Poverty, further disrupted family and community structures, resulting in profound psychological stress among ghetto inhabitants [10, 11].

These historical events illustrate the extraordinary trauma endured by Holocaust survivors. Trauma refers to events or series of events that involve life-threatening danger to an individual or their loved ones, disrupting the normal course of life and overwhelming one’s ability to cope [12]. The Holocaust – the state-sponsored genocide of six million Jews and numerous others by Nazi Germany – represents a singular form of trauma given its ideological extremism, bureaucratic precision, and industrial-scale destruction aimed at an entire population. Holocaust survivors experienced prolonged periods of extreme stress, danger, and deprivation during their early to mid-life, conditions that could have long-lasting health implications.

Oral health is a critical component of overall health, quality of life, and well-being. Tooth loss can impair fundamental functions such as speaking and eating, and it can limit social interaction due to aesthetic and functional challenges. Significant tooth loss often leads individuals to avoid certain foods, potentially negatively affecting nutrition and general health. Perceptions of both general health and oral health tend to be worse in those with extensive tooth loss, and advanced age itself is associated with diminished self-rated health [13]. In clinical terms, the retention of at least 20 natural teeth is commonly regarded as the minimum threshold for functional dentition and is associated with better oral health outcomes in older adults [14].

The impact of wartime hardship on oral health is a multifaceted phenomenon where systemic stressors, nutritional deficiencies, and the collapse of healthcare infrastructure converge to create long-term pathologies. Research indicates that individuals exposed to conflict-related trauma exhibit significantly higher rates of dental caries, periodontal disease, and tooth loss compared to non-affected populations [15].

The primary driver of this decline is the disruption of preventive care cycles. During active conflict, dental services are often deprioritized or become inaccessible, leading to the progression of manageable conditions into acute infections. Furthermore, wartime environments are characterized by “food insecurity”, often resulting in a diet high in fermentable carbohydrates and low in essential micronutrients, which accelerates demineralization [16].

Beyond physical barriers, the psychobiological link plays a critical role. Chronic stress associated with displacement and violence elevates cortisol levels, which is linked to immunosuppression and an increased susceptibility to inflammatory oral diseases like periodontitis [17]. Additionally, many survivors develop “parafunctional habits” - such as bruxism (teeth grinding), as a somatic manifestation of Post-Traumatic Stress Disorder (PTSD), leading to significant occlusal wear and temporomandibular joint disorders [18].

The oral health status of war survivors is not merely a clinical concern but a social one. Poor oral health is strongly correlated with reduced quality of life, impaired nutrition, and social withdrawal due to aesthetic shame [19]. Addressing these disparities requires trauma-informed dental care that accounts for the psychological triggers survivors may face in a clinical setting.

This study aimed to evaluate the long-term impact of living under the Nazi regime on the oral health of Holocaust survivors approximately sixty years after World War II. We hypothesized that the unique traumatic exposures of Holocaust survivors would be associated with poorer oral health outcomes in late life, even decades after the events.

## Methods

### Study design and population

We conducted a cross-sectional analysis based on data from a national survey of older adults in Israel. The survey, carried out between July 2005 and December 2006 by the Israel Center for Disease Control in collaboration with the Ministry of Health, targeted individuals aged 65 and above. The study population for our analysis consisted of Jewish Israeli citizens, including Holocaust survivors (HS) and a comparison group of Jewish peers who did not experience the Holocaust.

A stratified sampling approach was used. Initial sampling frames were obtained from the two largest health maintenance organizations in Israel, stratified by locality and population group (Jewish or Arab). The first random sample included 5,100 individuals. Due to a lower than expected response rate, a second sample of 4,250 individuals (restricted to Jews) was drawn to augment the study population. Inclusion criteria for the Holocaust survivor group were: (1) having lived under Nazi occupation during World War II (e.g., in a ghetto, labor camp, concentration camp, or in hiding) such that the individual’s life was in danger; (2) having lived in a country influenced by the Nazi regime (e.g., Romania, France, Italy, Hungary) and suffered persecution; or (3) having fled Nazi-controlled areas (e.g., Germany or Austria after 1938, or other affected countries after the war’s outbreak) up until May 8, 1945. Inclusion criteria for controls were being a Jewish Israeli of the same age group with no personal experience of Nazi persecution. Exclusion criteria for the survey were: residing in a hospital or psychiatric institution for more than six months, immigration to Israel after December 31, 2003 (to ensure a comparable lived experience in Israel), severe cognitive impairment precluding informed consent, or living in very remote areas that were logistically infeasible to survey.

### Data collection

Each selected individual was sent a cover letter explaining the survey and inviting participation. Approximately two weeks after the letters were mailed, trained interviewers attempted telephone contact. Up to eight call attempts were made to reach each person. Those who could be contacted and agreed to participate were scheduled for a face-to-face interview in their home. Interviews were conducted in the respondent’s preferred language (Hebrew, Arabic, Russian, or English) by one of 15 specially trained interviewers. If an individual could not be reached by phone after repeated attempts or refused participation when contacted, they were recorded as non-responders.

In accordance with the Declaration of Helsinki ethical approval was obtained from the Israeli Ministry of Health Ethics Committee.

All participants provided written informed consent to participate. The informed consent process included permission to conduct various health assessments (e.g., blood and urine tests and an oral examination) and for the collected data to be retained in a research database. During the in-home interview, a structured questionnaire was administered. The questionnaire gathered information on a wide range of topics, including demographic characteristics, general health status and medical history, oral and dental health status, functional status (e.g., activities of daily living), cognitive and psychological status, use of medications and dietary supplements, alcohol consumption, physical activity, smoking habits, dietary and eating habits, and knowledge and attitudes regarding nutrition. Key oral health variables recorded were the presence of natural teeth, use of dentures (partial or full, upper and/or lower), and any noted oral health problems.Following the interview, a brief standardized oral examination was performed for those participants who consented (1459 participants consented to the oral exam). Licensed dentists, trained and calibrated for the survey, conducted the oral examinations using the World Health Organization standard oral health assessment form. The oral exam documented the condition of the teeth and oral cavity, including number of decayed, missing, and filled teeth, presence of dentures, signs of periodontal disease (such as gum inflammation or tartar), and any observable oral lesions (such as suspicious lesions for oral cancer). Relevant findings from these oral examinations are included in our analysis, particularly the edentulism status and denture usage.

Efforts were made to evaluate the representativeness of the survey respondents and to identify potential non-response bias. A brief telephone follow-up survey was conducted on a subset of individuals who either could not be reached or refused participation initially. This follow-up gathered basic demographic data (age, marital status, religion, level of religiosity, education, and income) and health information (self-reported health conditions, functional status, smoking status, height, and weight for BMI calculation). Additionally, a 10% sample of those who refused was asked about their primary reasons for refusal. The most commonly cited reasons for non-participation were lack of time and personal illness (either of the invitee or their spouse).

The overall response rate for the survey was modest. In the first sampling wave, approximately 29.7% of those sampled completed the interview, and in the second wave the response rate was 28.2%. The combined response rate was roughly one-third of those originally contacted. The follow-up comparison between respondents and non-respondents revealed no major differences in basic demographic and health indicators, suggesting that, despite the relatively low response rate, the interviewed sample was not profoundly biased in those characteristics. Nonetheless, the response rate and potential selection bias are considered in interpreting the results (and are further addressed in the Discussion).

### Data processing and statistical analysis

Survey data were entered and cleaned using SAS software (version 9.0; SAS Institute, Cary, NC, USA). Statistical analyses were performed using SPSS software (version 28.0; IBM Corp.). Descriptive statistics were used to characterize the study population. Bivariate analyses were conducted to compare Holocaust survivors and controls on key variables: continuous variables were compared using Student’s t-tests, and categorical variables were compared using Chi-square tests (or Fisher’s exact test when appropriate).

To identify independent predictors of poor oral health outcomes, we carried out a multivariate logistic regression analysis. The primary outcome of interest was edentulism with prosthetic rehabilitation, operationalized as having two full dentures (upper and lower). This outcome was chosen as a proxy for severe oral health impairment (complete tooth loss). Predictor variables considered in the model included demographic factors (age, sex, education), health behaviors (smoking status, dental care utilization), socioeconomic indicators (pension receipt, having a caregiver), religiosity level, and Holocaust exposure (survivor vs. control). All variables that showed a significant association with the outcome in univariate analyses were entered into the multivariable logistic model. The logistic regression used a enter method (simultaneous inclusion of predictors) to estimate odds ratios (OR) and 95% confidence intervals (CI) for each predictor. Model fit was summarized by the Nagelkerke pseudo R-square. A two-tailed p-value < 0.05 was considered statistically significant for all analyses.

## Results

In the statistical processing for the purpose of this study, 1459 Jews were included, among them 396 Holocaust survivors, of which 194 were men and 202 were women.

Multivariate logistic regression was used to test the effects of various independent variables on the dependent variable: prevalence of two full dentures, all significant predictors were entered into a multiple logistic regression analysis. Multivariate regression was performed in order to avoid potential confounders or modification or mediation of the variables that may affect the relationship and to standardize the relationship between the independent variables and the dependent variable.

The response rate in the first sample was 29.7% and in the second 28.2%. The average interview time was one hour and 40 min. About 70% of the interviews were conducted in Hebrew, about 14% in Russian and 0.1% in English.

Table 1 shows the gender distribution of the study population.

**Table 1:** Gender distribution

		Total N (%)	HS N (%)	Control N (%)	p-Value*
Gender	Male	679 (46.5)	194 (48.8)	489 (45.7)	0.31
	Female	780 (53.5)	202 (51.2)	581 (54.3)	

*HS* holocaust survivor *Chi-square test

Table 2 shows the distribution of living arrangement, level of religiosity, marital status, caregiver, receipt of a pension and smoking status. Although the majority of members of both groups resided in apartment buildings, 81% HS and 74% controls, the difference was statistically significant (*p* < 0.05). About 60% of HS defined themselves as non-religious, compared to about 51% of controls. There were similar divorce and marriage rates in both groups. Significantly more HS had a care giver and a pension. 7% vs. 3% of controls (*p* < 0.05) had a care giver and 65% vs. 53% of controls (*p* < 0.01) had pension respectively. 40.9% of the HS smoked in the past compared to 34.6% of controls (*p* = 0.047). There was missing data: residence of 15 HS and 13 controls; presence of a care giver 2 participants; pension payments 3 participants.

**Table 2:** Place of dwelling, religious level, marital status and smoking status of participants

		Total *N* (%)	HS *N* (%)	Control *N* (%)	*p*-Value*
Place of dwelling	Private home	273 (19.1)	55 (14.4)	218 (20.8)	0.02
	Apartment building	1086 (75.9)	307 (80.6)	779 (74.2)	
	Institution	72 (5.0)	19 (5.0)	53 (5.0)	
Religious level	Secular	773 (53.1)	229 (59.0)	544 (50.9)	0.04
	Traditional	480 (33.0)	120 (30.9)	360 (33.7)	
	Religious	184 (12.6)	32 (8.2)	152 (14.2)	
	Orthodox	12 (0.8)	3 (0.8)	9 (0.8)	
	Other	7 (0.5)	4 (1.0)	3 (0.3)	
Marital status	Single	45 (3.1)	10 (2.6)	35 (3.3)	0.41
	Married	922 (63.3)	234 (60.2)	688 (64.5)	
	Divorced	71 (4.9)	19 (4.9)	52 (4.9)	
	Widow	418 (28.7)	126 (32.4)	292 (27.4)	
Caregiver	Yes	62 (4.3)	26 (6.7)	36 (3.4)	0.01
	No	1395 (95.7)	362 (93.3)	1033 (96.6)	
Pension	Yes	818 (56.2)	254 (65.3)	564 (52.9)	< 0.01
	No	637 (43.8)	134 (34.4)	503 (47.1)	
Smoker	Yes	137 (9.4)	39 (10.1)	98 (9.2)	0.047
	Past	526 (36.3)	158 (40.9)	368 (34.6)	
	No	587 (54.3)	189 (49.9)	598 (56.2)	
Total **		1459 (100)	396 (27.1)	1063 (72.9)	

*HS* holocaust survivor *Chi-square test

Table 3 shows the distribution of the study population according to age, years of education and BMI. The mean age of the HS was significantly older than controls (*P* < 0.05). No significant difference was found between the groups regarding years of education or BMI (Table 3).

**Table 3:** Age, years of education and BMI of participants

	HS	Control	
	Mean ± SD	Mean ± SD	*p*-Value*
Age	78.90 ± 6.46	76.88 ± 6.31	0.02
Years of Education	12.28 ± 16.88	12.67 ± 18.87	0.15
BMI	27.30 ± 4.52	27.12 ± 4.16	0.25

*HS* holocaust survivor *Independent t-test

Description of dental variables of the population

There were no differences in percentage of participants that had been to the dentist up to 24 months prior to the survey. 49% of the study popualtion had full dentures, 56% of HS had two full dentures, whereas 54% of controls had one complete prosthesis or no prostheses (*p* < 0.01) (Table 4).

**Table 4:** Distribution of the study population, last dental visit and type of dental prosthesis

		Total *N* (%)	HS *N* (%)	Control *N* (%)	*p*-Value*
Timing of dental visit	0–6 months	578 (39.9)	150 (38.7)	428 (40.4)	0.87
	7–12 months	151 (10.4)	44 (11.3)	107 (10.1)	
	13–24 months	134 (9.3)	37 (9.5)	97 (9.2)	
	> 24 months	585 (40.4)	157 (40.5)	428 (40.4)	
Dentures	Two	705 (48.5)	218 (56.3)	487 (45.7)	< 0.01
	One or none	748 (51.5)	169 (43.7)	579 (54.3)	
Total		1453 (100.0)	387 (26.6)	1066 (73.4)	

*HS* holocaust survivor *Chi-square test

From Table 5 it is evident that age (numerical), smoking (in the past or now versus no smoking at all), date of last dental visit, living under the Nazi regime (yes/no), level of religiosity (religious - An individual who leads a lifestyle guided by religious faith and a commitment to *mitzvot* (commandments), such as observing the Sabbath, keeping Kosher, and regular prayer. /non-religious) and help from a caregiver were statistically significant predictors of having two complete dentures. The independent variable age after dichotomization according to the median (76 years) was found to be a protective factor (OR = 0.42) for two full dentures, i.e., being younger was a protective factor against two complete dentures. Current or past smoking was a risk factor for having two full dentures (OR = 1.707). Those visiting the dentist within a year of the survey had a greater the chance of having two full dentures (OR = 3.725), as was being under the Nazi regime, and having a caregiver (OR = 1.905). In contrast, the level of religiosity was protective against dentures (*p* < 0.001) and marital status had no impact.

**Table 5:** Multiple logistic regression of variables related to having 2 full dentures (R-square = 0.222)

		B	Std. error	OR	95% confidence interval		Sig
					Lower bound	Upper bound	
Age –median 76	Below 76 vs. above	− 0.864	0.122	0.421	0.332	0.535	< 0.01
Smoke	Yes (past + current) vs. No	0.535	0.204	1.707	1.144	2.547	0.029
Last dental visit	0–12 months vs. >12 month	1.315	0.117	3.725	2.960	4.636	< 0.01
Under Nazi regimen	Yes vs. No	0.409	0.162	1.505	1.096	2.065	0.012
Religious level	Religious vs. Not religious	-0.410	0.118	0.664	0.527	0.836	< 0.01
Marital status	Married vs. Not married	0.216	0.124	1.241	0.974	1.582	< 0.01
Caregiver assistance	Yes vs. No	0.645	0.316	1.905	1.026	3.538	0.04
Constant		-0.473	0.142	0.623			< 0.01

## Discussion

The physiological and psychological consequences of the Holocaust have been widely studied [20]. The findings of the present study demonstrate that Holocaust survivors exhibit a significantly higher prevalence of complete edentulism compared to their peers. This observation aligns with the broader understanding of how extreme hardship and systemic neglect during conflict-oriented periods manifest as long-term oral pathologies. While no prior study has specifically isolated the oral health of this cohort, our results resonate with established research regarding the physiological and psychological “scarring” associated with the Holocaust [21, 22].

Data regarding the health status of 1,852 Israeli residents aged 65+, were collected, and only data from the 1,459 Jews were included in the current study. To maintain coherence of the research groups, the 393 Israeli Arabs were excluded because of inherent cultural diversity, customs and traditions that distinguish them from Jews and may bias the results of the study [21].

We focused on the presence of two full dentures as a proxy for complete tooth loss, which reflects the most severe form of oral health deterioration, often representing cumulative damage accumulated over a lifetime.

Our analysis revealed that Holocaust survivors had significantly worse oral health outcomes compared to non-survivors of similar age. Over half of the survivors had lost all their natural teeth and relied on complete dentures—markedly higher than among the control group. The observed disparity in tooth loss is consistent with literature linking early-life starvation to adult chronic disease. Previous research has demonstrated that Holocaust survivors suffer from increased rates of obesity, hypertension, and diabetes [23, 24]. Our finding that further supports the “cascade of frailty” hypothesis.

This difference remained statistically significant even after adjusting for other variables in a multivariate model. The experience of surviving the Holocaust—which entailed extreme hardship, prolonged undernutrition, and chronic psychological trauma—appears to have had long-lasting effects on survivors’ oral health, evident even six decades later.

Survivors in our sample were, on average, approximately two years older than the control group. Since tooth loss accumulates with age, this small difference could partly explain higher denture rates among survivors. Indeed, age was a strong predictor in our regression model: participants younger than 76 were significantly less likely to have complete dentures than those older than 76. This likely reflects generational improvements in dental care. However, the relatively small age difference does not fully account for the observed disparity, suggesting additional underlying causes.

Smoking history emerged as another key explanatory factor. Consistent with existing literature, smoking is strongly associated with periodontal disease, dental caries, and subsequent tooth loss. In our study, survivors had a higher proportion of former smokers compared to the control group. Our regression model showed that smokers were approximately 1.7 times more likely to have complete dentures than no smokers. This aligns with prior studies linking smoking to both oral and systemic health risks [25–27]. It is plausible that the trauma experienced during and after the Holocaust contributed to higher smoking rates among survivors, potentially as a coping mechanism. Regardless of the cause, smoking remains a modifiable risk factor. Its disproportionate prevalence among survivors underscores the importance of targeted cessation efforts and oral health prevention strategies in populations exposed to trauma.

Beyond behavioral factors, the physical effects of starvation and malnutrition likely contributed to survivors’ compromised oral health. Previous research has linked early-life deprivation among Holocaust survivors to increased prevalence of chronic diseases such as obesity, hypertension, diabetes, and hyperlipidemia [9]. These findings are particularly prominent among those who were children during the Holocaust, including those born during or shortly after the war. Our findings are consistent with this literature: osteoporosis, a condition influenced by long-term calcium and vitamin D deficiency, was significantly more common among survivors in our cohort (49.1% vs. 30.9% in controls, *p* < 0.01).

Osteoporosis is directly relevant to oral health. Deterioration of alveolar bone, the bony structure supporting teeth, can lead to tooth loss or hinder efforts to retain teeth into old age. Moreover, treatments for osteoporosis, particularly long-term bisphosphonate therapy, carry a risk of osteonecrosis of the jaw (ONJ), a serious complication [28, 29]. ONJ risk is heightened among older adults, especially those with a history of smoking, periodontal disease, diabetes, or complete edentulism [30–33]. Thus, we hypothesize a cascade: Holocaust-related early malnutrition increases osteoporosis risk, which in turn contributes to tooth loss and denture reliance, potentially compounding oral complications. While we did not measure ONJ directly, the conditions associated with its development were more common in survivors, warranting further investigation.

A notable and initially counterintuitive finding was that recent dental visits (within the past year) were associated with higher odds of having two full dentures. We interpret this not as a causal relationship, dental visits leading to edentulism, but rather as a reflection of denture users’ need for ongoing prosthetic maintenance.

For individuals with two full dentures, visits often reflect the necessity for prosthetic maintenance, such as adjustments to address mucosal irritation, relining of the denture base to compensate for ongoing alveolar bone resorption, or the management of denture-related stomatitis, which affects between 15% and 70% of wearers [34, 35]. Consequently, what may appear as a higher frequency of care is often a response to the mechanical and biological complications inherent to long-term prosthetic use. This interpretation is further supported by professional guidelines, such as those from the American College of Prosthodontists, which emphasize that even edentulous patients require annual professional evaluations to screen for oral cancer and ensure optimal prosthetic fit to prevent further tissue damage [36]. Rather than indicating a causal link where visits lead to tooth loss, these data suggest that the state of being edentulous creates a specific, recurring demand for clinical intervention to maintain oral function and quality of life.

One intriguing protective factor we identified was religiosity. Holocaust survivors who self-identified as religious or orthodox (characterized by a strict commitment to Halakha - Jewish Law) were less likely to have full dentures than their less religious counterparts. In the broader sample, religiosity was inversely associated with complete tooth loss. This supports findings from other studies linking religiosity and spirituality with improved health behaviors and outcomes [37, 38]. Religious individuals may benefit from stronger social support networks, healthier lifestyles, and coping mechanisms that reduce stress and promote health maintenance. Additionally, religion can offer a sense of purpose and routine, both of which may reinforce consistent self-care. While our data cannot confirm causation, the association is noteworthy. Survivors who embraced religious life after the war may have found community and structure that bolstered their health, including oral hygiene.

Another variable significantly associated with edentulism was the presence of a caregiver. Participants requiring caregiver assistance were more likely to have lost all their natural teeth. This is not surprising, as the need for caregiving often indicates declining general health or functional limitations. Such individuals may face challenges in maintaining oral hygiene or accessing dental services. Our findings highlight a critical consideration for geriatric care: individuals reliant on caregivers may need additional oral health support to prevent deterioration and ensure adequate prosthetic function.

The Holocaust survivors in our cohort experienced a uniquely set of circumstances, including physical deprivation, emotional trauma, loss of family, and long-term displacement [10, 22, 39]. Numerous studies have established that survivors face a higher burden of chronic illness and mental health challenges. Our study adds oral health to this list of long-term consequences. Complete edentulism can be viewed as another facet of the cumulative health burden endured by survivors, shaped by both early-life trauma and lifelong disadvantage.

### Limitations of the study

The findings of this study offer important insights into the intersection of historical trauma and geriatric oral health; however, they must be interpreted in light of several methodological limitations.

Response Rate and Selection Bias: A primary concern is the response rate of approximately 33%, which may introduce significant selection bias. In geriatric research, a lower response rate often results in a “healthy volunteer” bias, where individuals who are more functional, cognitively intact, and mobile are more likely to participate. Conversely, the most frail, institutionalized, or highly traumatized elderly individuals, who likely experience the poorest oral health and the most severe long-term effects of trauma, may be underrepresented. This potential exclusion suggests that our findings might actually underestimate the true prevalence of edentulism and oral health disparities among Holocaust survivors. Furthermore, we cannot rule out the possibility that those who declined to participate did so due to the sensitive nature of the study, potentially skewing the data toward survivors with higher levels of resilience or better social support systems.

Study Design and Causality: The cross-sectional nature of this study precludes the establishment of direct causal links between Holocaust exposure and subsequent tooth loss. We are effectively observing a “snapshot” of health that reflects a lifetime of cumulative biological and social factors rather than a documented progression of disease over time. While the associations remain robust in our models, they should be viewed as correlative.

Measurement and Heterogeneity: While the presence of two full dentures is a valid proxy for edentulism in this demographic, this measure may overlook “unmet needs”, individuals who are edentulous but lack access to or cannot afford prosthetics. Additionally, there is inherent heterogeneity within both the survivor and control groups. Survivors were analyzed as a single cohort, yet the specific toll of concentration camps likely differs from the experiences of those who survived in hiding or through forced labor. Similarly, the control group is subject to the “Healthy Migrant Effect”, as differences in country of origin, early-life nutrition, and access to preventive dental care across various geographical regions may introduce residual bias.

Confounding Factors: Finally, although we adjusted for key variables such as age and smoking, some residual confounding remains possible. Our data did not capture lifelong dietary habits (such as long-term sugar intake) or specific oral hygiene rituals across the life course, both of which are critical determinants of periodontal health and tooth retention.

Despite these limitations, our findings are consistent with broader evidence that Holocaust survivors carry a higher burden of chronic health problems. That this burden extends to oral health, and manifests as a significantly higher rate of complete tooth loss, is both clinically and ethically significant. Many survivors are now in their 80s and 90s, and ensuring that they receive adequate dental care, prosthetic support, and assistance with oral hygiene is an important priority. Policymakers and healthcare providers must recognize the unique needs of this population and allocate resources accordingly.

In summary, our study underscores the long-term oral health consequences of extreme early-life trauma. Holocaust survivors were significantly more likely to experience complete edentulism, even decades after the war. This disparity cannot be fully explained by age, smoking, or other demographic factors. Instead, it likely reflects the complex and lasting impact of prolonged deprivation, malnutrition, and psychological stress on the human body-particularly on oral health. These insights should inform efforts to improve dental care access, prevention strategies, and geriatric oral health services for trauma-exposed populations.

## Conclusion

Holocaust survivors carry a of long-term health challenges that extend to oral health. Recognizing and addressing these challenges is a matter of honoring their experiences with appropriate care and support as they age.

## Data Availability

The datasets analyzed during the current study were provided by the Israeli Ministry of Health and are not publicly available due to data sharing agreements, but may be  available from the corresponding author upon reasonable request and with permission from the Ministry of Health.

## List of abbreviartions

HS: Holocaust Survivors
BMI: Body Mass Index
OR: Odds Ratio
CI: Confidence Intervals
ONJ: Osteo Necrosis of the Jaw
